# Efficacy and Safety of Semaglutide for the Management of Obese Patients With Type 2 Diabetes and Chronic Heart Failure in Real-World Clinical Practice

**DOI:** 10.3389/fendo.2022.851035

**Published:** 2022-06-24

**Authors:** Luis M. Pérez-Belmonte, Jaime Sanz-Cánovas, María D. García de Lucas, Michele Ricci, Beatriz Avilés-Bueno, Lidia Cobos-Palacios, Miguel A. Pérez-Velasco, Almudena López-Sampalo, M. Rosa Bernal-López, Sergio Jansen-Chaparro, José P. Miramontes-González, Ricardo Gómez-Huelgas

**Affiliations:** ^1^Servicio de Medicina Interna, Hospital Regional Universitario de Málaga, Instituto de Investigación Biomédica de Málaga (IBIMA), Universidad de Málaga (UMA), Málaga, Spain; ^2^Servicio de Medicina Interna, Hospital Hospital Helicópteros Sanitarios, Marbella, Spain; ^3^Centro de Investigación Biomédica en Red Enfermedades Cardiovasculares (CIBERCV), Instituto de Salud Carlos III, Madrid, Spain; ^4^Servicio de Medicina Interna, Hospital Costa del Sol, Marbella, Spain; ^5^Servicio de Nefrología, Hospital Costal del Sol, Marbella, Spain; ^6^Centro de Investigación Biomédica en Red Fisiopatología de la Obesidad y Nutrición (CIBERobn), Instituto de Salud Carlos III, Madrid, Spain; ^7^Servicio de Medicina Interna, Hospital Universitario Rio Hortega, Departamento de Medicina, Facultad de Medicina, Universidad de Valladolid, Valladolid, Spain; ^8^Instituto de investigaciones biomédicas de Salamanca (IBSAL), Salamanca, Spain

**Keywords:** obesity, type 2 diabetes, heart failure, semaglutide, health status

## Abstract

**Background:**

The impact of glucagon-like peptide-1 receptor agonists on patients with heart failure has not been fully described. Our main objective was to evaluate the safety and clinical and glycemic efficacy of once-weekly semaglutide in obese patients with type 2 diabetes and heart failure.

**Methods:**

In this observational, retrospective, real-world study, we enrolled outpatients with type 2 diabetes, obesity, and heart failure who started semaglutide and were followed-up on at 3, 6, and 12 months.

**Results:**

A total of 136 patients were included. From baseline to 12 months, there was a significant improvement on the Kansas City Cardiomyopathy Questionnaire total symptom score (59.0 ± 24.1 vs 79.9 ± 28.4 points, p<0.01), a reduction in the proportion of patients with New York Heart Association functional class III (40.4% to 16.2%, p<0.01), and a reduction in N-terminal pro-brain natriuretic peptide levels (969.5 ± 653.5 vs 577.4 ± 322.1 pg/mL, p<0.01). Emergency department visits due to heart failure, hospitalizations due to heart failure, and all-cause hospitalizations also declined. Additionally, significant reductions in glycated hemoglobin (-1.4%) and body weight (-12.7 kilograms) were observed as well as a de-intensification of antidiabetic therapy. Moreover, semaglutide was safe and well-tolerated.

**Conclusion:**

In obese patients with type 2 diabetes and heart failure, the use of once-weekly semaglutide was safe and clinically efficacious, improving health and functional status. Nevertheless, more strong evidence on glucagon-like peptide-1 receptor agonists in heart failure is required.

## Introduction

In recent years, heart failure (HF) has become the most common cardiovascular disease in patients with type 2 diabetes (T2D); its incidence is 2.5 times greater than acute coronary disease (1). In addition, HF is among the most common causes of hospital admissions (2) and hospitalization rates for this disease are exponentially higher than for coronary artery disease and stroke (3). Patients with concomitant HF and T2D have a lower quality of life, worse symptoms and higher mortality compared to patients without T2D (4).

The mail clinical guidelines published by scientific societies in Europe, the United States of America, and Canada have made important changes to recommendations on the choice of treatment and management approach for patients with T2D. They recommend the preferential use of glucagon-like peptide-1 (GLP-1) receptor agonists and sodium−glucose cotransporter 2 (SGLT-2) inhibitors that have proven renal and cardiovascular benefits for patients with established cardiovascular disease or those at high risk for cardiovascular disease if there are no contraindications for their use (5–8).

Safety concerns, comorbidities, or their healthcare environment may affect many patients’ selection of glucose-lowering drugs (5). Most patients require intensification of glucose-lowering drugs. For those with T2D and HF -especially HF with reduced ejection fraction- or those who are at high risk of developing HF, chronic kidney disease, evident atherosclerotic cardiovascular disease, or any combination of these diseases, the use of SGLT-2 inhibitors with demonstrated cardiovascular benefit as an add-on metformin is recommended. However, GLP-1 receptor agonists with proven cardiovascular benefits have been recommended as the treatment of choice for patients with established atherosclerotic cardiovascular disease or patients who are at very high risk for developing it. Given the higher degree weight loss that has generally been reported with GLP-1 receptor agonists, their use is recommended for patients who are prioritizing weight loss or weight maintenance (5–8). In regards to HF outcomes, GLP-1 receptor agonists have not been associated with significant improvements in pivotal cardiovascular outcomes clinical trials (9–16). Nevertheless, when patients in all GLP-1 receptor agonists’ trials were analysed in a meta-analysis conducted by Kristensen et al. (17), use of GLP-1 receptor agonists was associated with a significant reduction in HF hospitalizations.

Despite this evidence, the impact of GLP-1 receptor agonists in patients with HF has not been fully described in the literature. In this study, our main objective was to evaluate the safety and clinical and glycemic efficacy of once-weekly semaglutide in obese patients with T2D and chronic HF during 12 months of follow-up. We also evaluated weight loss and the de-intensification of T2D and HF treatments after the initiation of semaglutide. We hypothesize that the initiation of semaglutide would have beneficial effects on clinical outcomes and glycemic control and is safe in patients with obesity, T2D and chronic HF.

## Materials and Methods

We conducted an observational, retrospective, real-world study on obese outpatients with T2D and chronic HF at the HF Units of Internal Medicine Department at the Hospital Regional Universitario de Málaga in Málaga; the Hospital Costal de Sol, in Marbella; and the Hospital Helicopteros Sanitarios, in Marbella, Spain.

Each patient’s electronic medical record was reviewed by investigators to gather patient data. The study was approved by the Institutional Research Ethics Committee of Málaga and written informed consent for the consultation of patient medical records was obtained from all participants. This study was conducted in accordance with the Declaration of Helsinki.

### Patients and Study Design

Patients started with a once-weekly dose of 0.25 mg for 4 weeks that could be increased to 0.5 mg for the following 4 weeks until they reached the maintenance dose (0.5 mg or 1.0 mg) provided that the healthcare professionals deemed it advisable according to their clinical judgment. During the follow-up, all patients received general recommendations on a healthy diet and physical activity according to their functional class. Treatment with diuretics, antihypertensive agents, and lipid-lowering drugs were modified, if necessary, as per the healthcare professionals’ judgment.

Follow-up was conducted at 3, 6, and 12 months after starting semaglutide. Data on a multitude of anthropometric (body weight, body mass index (BMI), and waist circumference), sociodemographic, clinical (T2D duration and treatment, principal cause of HF, HF duration, left ventricular ejection fraction, previous medical history, and medication), therapeutic (any reduction in the number or doses of T2D and HF drugs), and laboratory variables (serum creatinine, estimated glomerular filtration rate measured using Chronic Kidney Disease Epidemiology Collaboration (CKD-EPI) formulae (18), basal fasting blood glucose (BG), glycated hemoglobin (HbA1c), LDL, HDL and total cholesterol, triglycerides, uric acid, hematocrit, N-terminal pro-brain natriuretic peptide (NT-pro-BNP), and urinary albumin/creatinine ratio) were gathered at each evaluation. The total symptom score on the Spanish version of the Kansas City Cardiomyopathy Questionnaire (KCCQ) (19) and New York Heart Association (NYHA) classification were used to estimate HF heath status. Adverse drug reactions, need to discontinue semaglutide due to adverse events, 3-point major adverse cardiovascular events (3P-MACE) (composite of nonfatal stroke, nonfatal myocardial infarction, and cardiovascular death), emergency department visit because of HF decompensation; hospitalizations (HF and all-cause), and mortality (due to cardiovascular or non-cardiovascular causes) after starting semaglutide were also recorded.

### Measured Outcomes

Our primary endpoint was to evaluate the clinical efficacy in the HF health status, as determined by the improvement in the total symptom score on the Spanish version of the Kansas City Cardiomyopathy Questionnaire (KCCQ) (19), reduction in the New York Heart Association (NYHA) classification, and reduction in NT-pro-BNP levels. Secondary outcomes included the glycemic efficacy, as determined by the reduction in HbA1c levels and the proportion of patients who achieved good glycemic control (HbA1c <7%) prior to starting semaglutide and at 3, 6, and 12 months; weight loss (changes in body weight, BMI, and waist circumference); de-intensification of T2D treatment (reduction in number of daily glucose-lowering drugs and/or insulin doses) and HF treatment (decline in number of antihypertensive agents, beta-blockers and diuretics); and the safety (adverse drug reactions, need to discontinue semaglutide due to adverse events, 3P-MACE, emergency department visit because of HF decompensation (from one year before initiation), hospitalizations (from one year before initiation), and mortality (from one year before initiation).

### Statistical Analyses

Quantitative variables are shown as means ± standard deviation whereas qualitative variables are shown as absolute values and percentages. Quantitative variables were compared using Student’s t-test and the repeated measures analysis of variance whereas qualitative variables were compared using Pearson’s chi-square and McNemar’s test. The Pearson correlation coefficient was calculated to estimate the linear correlations between variables. Statistical significance was defined as p<0.05. Statistical analyses were performed using SPSS Statistics forWindows, version 15.0.

## Results

A total of 136 obese patients with T2D and chronic HF were included in this study. Metformin was the most frequent oral glucose-lowering drug used among the patients (59.6%), followed by dipeptidyl peptidase-4 (DPP-4) inhibitors (39.7%) and SGLT-2 inhibitors (38.2%). Non-semaglutide GLP-1 receptor agonists were used in the 33.8% of the study patients. Basal insulin was used by 86 patients (63.2%) with a mean dose of 39.0 units per day and insulin combinations were used by 16 patients (13.8%) before starting semaglutide. Baseline sociodemographic, clinical, and treatment variables are shown in **Table 1**.

**Table 1:** Baseline sociodemographic and clinical-therapeutic characteristics.

Variables	n = 136
Sociodemographic characteristics	
Age (years)	72.6 ± 11.0
Male gender	74 (54.4%)
Diabetes characteristics	
Diabetes duration (years)	13.5 ± 7.4
Diabetes therapy Metformin Sulfonylurea DPP-4 inhibitorGLP-1 receptor agonist (no semaglutide) SGLT-2 inhibitor Basal insulin Basal insulin dose (Units/day) Insulin combinations	81 (59.6%) 4 (2.9%) 54 (39.7%) 46 (33.8%) 52 (38.2%) 86 (63.2%) 39.0 ± 21.8 16 (13.8%)
Statins	122 (89.7%)
Heart failure characteristics	
Heart failure duration (years)	6,5 ± 3.2
Principal cause of heart failure Ischemic Non-ischemic	56 (41.2%) 80 (58.8%)
Left ventricular ejection fraction (%)	52.5 ± 10.9
Left ventricular ejection fraction <40%	54 (39.7%)
Heart failure medication Diuretic ACE inhibitor ARB Sacubitril-valsartan Beta-blocker Ivabradin Mineralocorticoid receptor antagonist Digitalis Anticoagulant	134 (98.8%) 24 (16.9%) 62 (45.6%) 34 (25.0%) 102 (75.0%) 25 (18.3%) 59 (43.4%) 16 (11.8%) 72 (52.9%)
Previous medical history	
History of smoking	76 (55.9%)
History of alcohol abuse	13 (9.6%)
Hypertension	136 (100.0%)
Dyslipidaemia	124 (91.2%)
Chronic kidney disease stage ≥3	88 (64.7%)
Cerebrovascular disease	15 (11.0%)
Chronic obstructive pulmonary disease	34 (25.0%)
Atrial fibrillation	62 (45.6%)

Continuous data are shown as means (standard deviations) and qualitative data as absolute value and percentage.

ACE, angiotensin-converting enzyme; ARB, angiotensin receptor blocker; DPP4, dipeptidyl peptidase-4; GLP-1, glucagon-like peptide-1; SGLT-2, sodium−glucose cotransporter 2.

All patients started with an initial once-weekly dose of 0.25 mg for 4 weeks except for patients who previously received GLP-1 receptor agonists, who were switched to 0.50 mg of semaglutide. Once-weekly semaglutide was increased to 1.00mg in 76 patients (59.8%) at 3 months, 93 patients (74.4%) at 6 months, and 100 patients (80.6%) at 12 months.

From baseline to 12 months, there was a significant improvement in the KCCQ total symptom score, which increased from 59.0 points to 79.9 points (p<0.01), as well as a significant reduction in the proportion of patients with NYHA functional class III, which declined from 40.4% to 16.2% (p<0.01), and NT-pro-BNP levels, which declined from 969.5 ± 653.5 to 577.4 ± 322.1 pg/mL (p<0.01). The KCCQ total symptom score correlated negatively with the NT-pro-BNP levels during the follow-up (r = -0.687, p<0.01).

In regard to glycemic control, a significant reduction in fasting BG and HbA1c were observed after initiating semaglutide. The proportion of patients with HbA1c <7% increased from 16.2% at baseline to 64.5% at 12 months (p<0.001).

During the follow-up, patients had a significant reduction in body weight (12.7 kg) and BMI (7.1 kg/m^2^), with a decline in the proportion of patients with obesity (BMI ≥30) to 50.8%. There were also significant reductions in the waist circumference and systolic blood pressure. A positive correlation was found between the body weight loss and the KCCQ total symptom score (r = 0.612, p<0.01).

Patients who started once-weekly semaglutide experienced a de-intensification in their T2D treatment from baseline to 12 months’ follow-up, with declines in the number of daily glucose-lowering drugs (3.5 ± 1.2 to 2.2 ± 0.8, p<0.05), the proportion of patients with basal insulin (63.2% to 32.3%, p<0.001), basal insulin dose (39.0 ± 21.8 to 20.2 ± 8.9, p<0.001), and the proportion of patients with insulin combinations (13.8% to 0, p<0.01). No differences were observed in HF medications.

A significant difference was found in the urinary albumin/creatinine ratio from baseline to 12 months, with a reduction of 39 mg/g. No changes were observed in other laboratory parameters. The urinary albumin/creatinine ratio correlated negatively with the KCCQ total symptom score during the follow-up (r = -0.528, p<0.01).

In regard to safety, 30 patients (24.2%) had adverse drug reactions (mostly gastrointestinal disorders) which led to discontinuation of semaglutide in 12 of them (11 patients due to gastrointestinal disorders). There were six 3P-MACEs linked to two cardiovascular deaths. Emergency department visits due to HF, hospitalizations due to HF, and all-cause hospitalizations were lower 12 months after starting semaglutide compared to the data reported in the 12 months before starting semaglutide (50.7% vs. 39.5%, p<0.05; 38.2% vs 27.4%, p<0.05; and 8.8% vs 4.0%, p<0.05; respectively).

All data on clinical efficacy in the HF health status, glycemic control, anthropometric characteristics, treatment de-intensification, laboratory variables, and safety are summarized in **Table 2**.

**Table 2:** Clinical efficacy in the heart failure health status, glycemic control, anthropometric characteristics, treatment de-intensification, laboratory variables, and safety.

Variables	Baseline (n = 136)	3 months’ follow-up (n = 127)	6 months’ follow-up (n = 125)	12 months’ follow-up (n = 124)
HF health status				
KCCQ total symptom score	59.0 ± 24.1	68.9 ± 25.1*	75.5 ± 27.5^†^	79.7 ± 28.4^†^
NYHA functional class I II III	0 81 (59.6%) 55 (40.4%)	0 88 (69.3%) *39 (30.7%)*	6 (4.8%) 94 (75.2%)^†^ 25 (20.0%)^†^	7 (5.6%) 97 (78.2%)^†^ 20 (16.2%)^†^
NT-proBNP (pg/mL)	969.5 ± 653.5	738.8 ± 553.4*	610.9 ± 424.5^†^	577.4 ± 322.1^†^
Glycemic control				
Fasting blood glucose (mg/dL)	154.9 ± 50.0	140.4 ± 42.2^†^	121.5 ± 38.4^‡^	117.0 ± 33.2^‡^
HbA1c (%)	8.1 ± 1.4	7.3 ± 1.2*	7.0 ± 1.1^†^	6.7 ± 1.0^†^
Patients with HbA1c <7%	22 (16.2)	45 (35.4%)*	66 (52.8%)^‡^	80 (64.5%)^‡^
Anthropometric characteristics				
Body weight (kg)	97.8 ± 19.7	92.8 ± 16.7*	87.1 ± 14.4^†^	85.1 ± 13.2^‡^
Body Mass Index (kg/m2)	36.6 ± 7.2	33.9 ± 6.5*	30.9 ± 4.9^†^	29.5 ± 4.3^‡^
Body Mass Index ≥30	136 (100.0%)	103 (81.1%)*	80 (64.0%)^†^	61 (49.2%)^‡^
Waist circumference (cm)	133.3 ± 19.1	119.1 ± 15.2*	108.8 ± 12.7^†^	102.9 ± 10.0^†^
SBP (mmHg)	130.9 ± 12.3	122.4 ± 11.1	119.2 ± 10.4*	118.4 ± 10.0*
DBP (mmHg)	72.5 ± 13.6	71.5 ± 12.8	69.1 ± 9.9	68.4 ± 8.9
Heart rate (bpm)	75.1 ± 12.6	72.4 ± 10.2	70.2 ± 8.9	69.7 ± 8.2
Treatment de-intensification				
Number of daily antidiabetic agents	3.5 ± 1.2	2.5 ± 0.9*	2.4 ± 0.9*	2.2 ± 0.8*
Basal insulin	86 (63.2%)	70 (55.1%)*	48 (38.4%)^‡^	40 (32.3%)^‡^
Basal insulin dose (Units/day)	39.0 ± 21.8	32.9 ± 18.5*	24.6 ± 11.0^†^	20.2 ± 8.9^‡^
Insulin combinations	16 (13.8%)	9 (7.1%)*	1 (0.8%)^†^	0^†^
Laboratory variables				
Creatinine (mg/dL)	1.2 ± 0.5	1.2 ± 0.5	1.3 ± 0.5	1.3 ± 0.5
EGFR (ml/min/1.73 m^2^)	56.8 ± 22.4	55.9 ± 21.2	56.4 ± 22.8	57.7 ± 22.9
Uric acid (mg/dL)	7.7 ± 5.0	7.1 ± 4.8	6.8 ± 3.8	6.7 ± 3.5
Hematocrit (%)	41.7 ± 5.5	43.0 ± 5.8	43.6 ± 6.3	43.7 ± 6.4
LDL cholesterol (mg/dL)	78.4 ± 28.5	77.4 ± 24.5	70.7 ± 21.1	71.5 ± 21.2
HDL cholesterol (mg/dL)	40.4 ± 9.5	39.8 ± 9.1	41.1 ± 10.5	43.1 ± 12.0
Total cholesterol (mg/dL)	158.7 ± 39.2	159.9 ± 39.5	147.6 ± 40.0	151.4 ± 44.4
Triglycerides (mg/dL)	203.9 ± 60.0	197.3 ± 56.4	200.5 ± 58.1	207.4 ± 59.1
Urinary albumin/creatinine ratio (mg/g)	52.4 ± 47.8	46.4 ± 40.8*	23.6 ± 12.8^†^	13.4 ± 9.9^‡^
Safety variables^a^				
Adverse drug effects	–	12 (9.4%)	25 (20.0%)	30 (24.2%)
Gastrointestinal disorder Nausea Vomiting Diarrhea	–	12 5 4 3	24 11 8 5	30 14 10 6
Acute pancreatitis	–	0	1	0
Discontinuation of semaglutide	–	9 (6.6%)	11 (8.1%)	12 (8.8%)
Major complication^a,b^				
3P-MACE	–	0	2 (1.6%)	6 (4.8%)
Emergency department visit because of HF	69 (50.7%)	20 (15.7%)	30 (24.0%)	49 (39.5%)*
Hospitalization Because of HF All-cause	52 (38.2%) 12 (8.8%)	10 (7.9%) 2 (1.6%)	19 (15.2%) 3 (2.4%)	34 (27.4%)* 5 (4.0%)*
Mortality Cardiovascular cause Noncardiovascular cause	–	0 0	0 0	2 (2.4%) 0
HF hospitalization and cardiovascular mortality	–	10 (7.9%)	19 (15.2%)	36 (29.0%)

Continuous data are shown as means (standard deviations) and qualitative data as absolute value and percentages. Statistical significance was measured for the comparison of baseline and follow-up data.

DBP, diastolic blood pressure; EGFR, estimated glomerular filtration rate; HbA1c, glycated hemoglobin; HF, heart failure; KCCQ, Kansas City Cardiomyopathy Questionnaire; 3P-MACE, 3-Point major adverse cardiovascular event; NT-proBNP, N-terminal pro-brain natriuretic peptide; NYHA, New York Heart Association; SBP, systolic blood pressure

a Cumulative data during the 12 months of follow-up are shown.

b Data from one year before initiation (baseline) were compared with 3, 6, and 12 months’ follow-up.

*p<.05.

^†^p<.01.

^‡^p<.001.

No significant differences were found in the KCCQ total symptom score, NT-pro-BNP levels, fasting BG, HbA1c, and body weight during the follow-up period according to estimated glomerular filtration rate (<60 vs ≥60 ml/min/1.73 m^2^. All these data shown in **Table 3**.

**Table 3:** Kansas City Cardiomyopathy Questionnaire total symptom score, N-terminal pro-brain natriuretic peptide levels, fasting blood glucose, glycated hemoglobin, and body weight according to estimated glomerular filtration rate.

Variables	EGFR	Baseline (n = 136)	3 months’ follow-up (n = 127)	6 months’ follow-up (n = 125)	12 months’ follow-up (n = 124)
KCCQ total symptom score	<60 ml/min/1.73 m^2^	58.1 ± 24.0	68.1 ± 24.8	74.9 ± 27.1	79.5 ± 28.3
	EGFR ≥60 ml/min/1.73 m^2^	59.9 ± 24.3	69.7 ± 25.4	76.1 ± 27.8	79.9 ± 28.5
p-value		0.187	0.201	0.198	0.298
NT-proBNP (pg/mL)	<60 ml/min/1.73 m^2^	949.5 ± 651.1	713.6 ± 547.9	598.6 ± 420.7	571.0 ± 318.9
	EGFR ≥60 ml/min/1.73 m^2^	989.1 ± 659.2	764.0 ± 563.4	623.2 ± 429.2	583.8 ± 326.2
p-value		0.164	0.101	0.111	0.179
Fasting BG (mg/dL)	<60 ml/min/1.73 m^2^	156.4 ± 50.8	140.8 ± 42.4	122.5 ± 38.6	118.5 ± 33.5
	EGFR ≥60 ml/min/1.73 m^2^	153.4 ± 49.3	140.0 ± 42.0	120.5 ± 38.0	115.5 ± 32.8
p-value		0.242	0.301	0.238	0.237
HbA1c (%)	<60 ml/min/1.73 m^2^	8.2 ± 1.5	7.4 ± 1.3	7.1 ± 1.1	6.9 ± 1.2
	EGFR ≥60 ml/min/1.73 m^2^	8.1 ± 1.4	7.2 ± 1.2	7.0 ± 1.1	6.7 ± 1.0
p-value		0.302	0.289	0.311	0.291
Body weight (kg)	<60 ml/min/1.73 m^2^	96.8 ± 19.2	92.3 ± 16.4	86.6 ± 14.2	84.6 ± 13.1
	EGFR ≥60 ml/min/1.73 m^2^	98.8 ± 19.9	93.3 ± 16.8	87.6 ± 14.5	85.5 ± 13.2
p-value		0.199	0.283	0.295	0.300

Continuous data are shown as means (standard deviations) and qualitative data as absolute value and percentages. Statistical significance was measured for the comparison of EGFR groups.

BG, blood glucose; EGFR, estimated glomerular filtration rate; HbA1c, glycated hemoglobin; KCCQ, Kansas City Cardiomyopathy Questionnaire; NT-proBNP, N-terminal pro-brain natriuretic peptide.

## Discussion

This study found that obese patients with T2D and chronic HF who were treated with once-weekly semaglutide improved their HF health status, increasing the quality of live and reducing the functional class and the NT-proBNP levels. Semaglutide was also efficacious in regard to glycemic control, with reductions in fasting BG and HbA1c levels and body weight. In addition, patients treated with semaglutide experienced a significant de-intensification of T2D treatment, with reductions in the number of daily glucose-lowering drugs, basal insulin doses, and proportion of patients with insulin therapy, with a good tolerability profile. Semaglutide was also associated with reductions in emergency department visits due to HF, hospitalizations due to HF, and all-cause hospitalizations.

The coexistence of chronic HF with T2D is common and T2D is considered one of the significant risk factor for adverse outcomes in patients with HF (2–4). In recent years, the cardiovascular safety of glucose-lowering drugs, especially with SGLT-2 inhibitors and GLP-1 receptor agonists, has become an important topic and several cardiovascular outcome trials have been performed. Whereas SGLT-2 inhibitors have clearly shown benefits in HF hospitalizations in patients with (20–24) and without T2D (25, 26), GLP-1 receptor agonists have not been demonstrated to lead to significant reductions in HF hospitalizations (9–16). However, when all patients from all GLP-1 receptor agonists’ trials were analysed in a meta-analysis carried out by Kristensen et al. (17), GLP-1 receptor agonists were associated with a significant reduction in HF hospitalizations. Recently, a systematic review of observational studies found conflicting results in terms of HF outcomes of GLP-1 receptor agonists, with some studies showing lower rates of HF hospitalization among GLP-1 receptor agonists’ users and other studies showing neutral effects on HF hospitalizations (27). One of these studies that showed benefits was a retrospective cohort of 1,426 users of GLP-1 receptor agonists and 2,798 control subjects. After a propensity score matched analysis, the use of GLP-1 drugs were associated with a risk reduction of 49% in HF hospitalization. There were also significant reductions in all-cause hospitalization (46%) and deaths (69%) (28). In another study that prospectively included 288 patients with T2D and chronic HF treated with cardiac resynchronization therapy with a defibrillator, the use of GLP-1 receptor agonists in addition to conventional hypoglycemic therapy was associated with a significant decline in NYHA class, higher scores on the 6-minute walking test, and a higher probability of the patients responding to cardiac resynchronization therapy with a defibrillator. Additionally, GLP-1 receptor agonist users experienced fewer arrhythmic events, a lower rate of hospitalization for HF worsening, and a higher probability of the patient responding to cardiac resynchronization therapy with a defibrillator in the follow-up period (29).

These benefits on HF outcomes are consistent with our results. In our study, the use of semaglutide was associated with improvement in HF health status, increasing the quality of live and reducing the functional class. As secondary outcomes, we also observed benefits associated with the use of semaglutide in reductions in emergency department visits due to HF, hospitalizations due to HF and all-cause hospitalizations.

Although at present there are not studies reporting benefits of once-weekly semaglutide on HF outcomes focused on patients with HF, in the SUSTAIN-6 trial -the pivotal cardiovascular outcome trial of subcutaneous semaglutide in patients with T2D and high cardiovascular risk (23.6% with HF)- no significant benefits were observed in hospitalizations for HF when compared with placebo group (11).

Several drugs routinely used in HF, such as angiotensin-converting enzyme inhibitors, angiotensin receptor-neprilysin inhibitor, beta-blockers, and mineralocorticoid receptor antagonists, have been associated with greater survival, a reduction in the risk of HF hospitalization, and a reduction in symptoms in patients with HF with reduced ejection fraction (30). Recently, SGLT-2 inhibitors have also shown robust benefits in HF hospitalizations in patients with HF with reduced ejection fraction regardless of the presence of T2D in the DAPA-HF (dapagliflozin) (25) and EMPEROR-Reduced (empagliflozin) (26) clinical trials. Furthermore, positive results have been shown in the EMPEROR-Preserved trial (31), a clinical trial on empagliflozin versus a placebo in patients with HF with preserved ejection fraction regardless of presence of T2D. Empagliflozin was associated with a significant decline in the composite variable of cardiovascular death or hospitalization due to HF. A recent meta-analysis also found a significant association between SGLT-2 inhibitors and an improvement in health-related quality of life (32).

In our study, we also observed a reduction of NT-proBNP levels with the use of semaglutide. This benefit has been previously observed in a study using liraglutide on patients with T2D and HF with reduced ejection fraction (33).

The benefits on HF outcomes observed with the use of GLP-1 receptor agonists may be due to their direct effects through actions on endothelial dysfunction and inflammation (34), reducing the circulating immune cell and chemokine levels, the vascular expression of pro-inflammatory mediators and leukocyte adhesion molecules, and the vascular infiltration by immune cells (35). GLP-1 receptor agonists have been shown to modulate water and sodium homeostasis, reducing hyperfiltration and increasing natriuresis (36). Other potential protective mechanisms include their effects on the renin-angiotensin system, principally a reduction in renin-angiotensin-aldosterone system activation markers (37). GLP-1 receptor agonists may have indirect cardiorenal protective benefits given their effects on improving blood pressure due to atrial natriuretic peptide release from atrial cardiomyocites and nitric oxide production (35), and glycemic control, increasing insulin sensitivity, and reducing insulin levels. The sustained weight loss associated with GLP-1 receptor agonists and the potential impacts on the gut microbiota composition may also be potential beneficial factors (34, 37). Furthermore, GLP-1 receptors have been found in the heart. The action of GLP-1 may favor myocardial glucose uptake regardless the insulin secretion *via*, ameliorating insulin resistance associated with HF though the up-regulation of GLP-1 isoforms such as sarcolemmal and endosome Glut4. GLP-1 cardiomyocite-independent actions may improve coronary flow and left ventricular wall motion, improving myocardial function (35, 38–40). The adipose tissue metabolome has been also described as modifiable regulators of vascular redox state in obese patients, directly impacting on cardiovascular outcomes in patients with established atherosclerosis (41). In addition, treatment with a GLP-1 receptor agonist such as liraglutide has been associated with a maintained reduction in apoB levels, potentially contributing to a lower cardiovascular risk (42). An endogenous GLP-1 response induced by oral glucose has been associated with clinically relevant lower central and peripheral blood pressures, which may also contribute to a decreased cardiovascular risk (43). In our study, the improvement in glycemic control (reduction of 1.4% in HbA1c at 12 months of follow-up) and reduction in body weight (weight loss of 12.7 kg at 12 months of follow-up) may also be potential factors involved in the benefits on the HF health status outcomes. Recently, a strategy combining moderate-to-vigorous-intensity exercise and liraglutide therapy led to greater weight loss than exercise or liraglutide alone in patients with obesity. The combination strategy was also associated with improvements in the HbA1c level, insulin sensitivity, and cardiorespiratory fitness (44). This finding shows the importance of implementing structured treatment programs using GLP-1 receptor agonists in combination with exercise in order to achieve long-term weigh loss goals and health benefits. Moreover, the use of insulin has been described as a drug that could worsen HF (45). We observed a significant reduction in the proportion of patients with insulin after starting once-weekly semaglutide. This finding may also contribute to the clinical benefit observed in obese patients with T2D and HF.

All these potential cardiovascular benefits in obese patients with T2D and HF could be also extrapolated to patients without T2D. Long-term efficacy and safety have been shown for GLP-1 receptor agonists as anti-obesity drugs (46).

Another relevant finding from our study was the de-intensification of the antidiabetic treatment, with fewer daily glucose-lowering drugs. Treatment simplification is especially important in patients with T2D and HF, who are normally subject to polypharmacy and more likely to have adverse drug reactions (45, 47). Simplification of the antidiabetic treatment have been recently shown with the SGLT-2 inhibitor canagliflozin in patients with HF and T2D, with significant reduction in number of glucose-lowering drugs, basal insulin dose, and percentage of patients who used basal insulin after switching non-insulin glucose lowering drugs (excluding metformin) to canagliflozin (48).

Though our results are important, this study has several limitations. First, the observational nature of our data, the limited number of patients, and the lack of a control group might have led to bias. Second, considering the low number of complications or events, their relationship to the use of semaglutide could not be conclusively determined. Third, as the HF medication could be modified if deemed advisable according to healthcare providers’ judgment together with the fact that patients were given general recommendations during follow-up on a healthy diet and physical activity suitable to their functional class, the entirety of our findings cannot be strictly attributed to initiating semaglutide. Finally, only once-weekly semaglutide was evaluated in our study. Due to these limitations, our findings cannot be extrapolated to other GLP-1 receptor agonists.

In conclusion, the use of once-weekly semaglutide improved HF health status, increasing quality of live and reducing functional class and NT-proBNP levels in obese patients with T2D and chronic HF. Semaglutide was also efficacious in regard to glycemic control and body weight reduction. In addition, patients treated with semaglutide experienced a significant de-intensification of T2D treatment, with a good tolerability profile. Reductions emergency department visits due to HF, hospitalizations due to HF, and all-cause hospitalizations were also observed. Randomized clinical trials with GLP-1 receptor agonists are required to provide more evidence on the efficacy and safety of GLP-1 receptor agonists in patients with HF.

## Data Availability Statement

The raw data supporting the conclusions of this article will be made available by the authors, without undue reservation.

## Ethics Statement

The studies involving human participants were reviewed and approved by Institutional Research Ethics Committee of Málaga. The patients/participants provided their written informed consent to participate in this study.

## Author Contributions

LP-B and JS-C contributed to the conception, design of the work the acquisition, interpretation of data, writing-original draft preparation, writing-review and editing, and supervision. MG, MR, BA-B, LC-P, MP-V, AL-S, MB-L, SJ-C, and JM-G contributed to the acquisition of data and revised the work. JS-C contributed to interpretation of data, writing-review and editing, and supervision. RG-H was a major contributor in interpretation of data, writing-original draft preparation, writing-review and editing, and supervision. All authors read and approved the final manuscript. All authors meet the criteria for authorship stated in the Uniform Requirements for Manuscripts Submitted to Biomedical Journals.

## Funding

This research received no specific grant from any funding agency in the public, commercial, or not-for-profit sectors.

## Conflict of Interest

The authors declare that the research was conducted in the absence of any commercial or financial relationships that could be construed as a potential conflict of interest.

## Publisher’s Note

All claims expressed in this article are solely those of the authors and do not necessarily represent those of their affiliated organizations, or those of the publisher, the editors and the reviewers. Any product that may be evaluated in this article, or claim that may be made by its manufacturer, is not guaranteed or endorsed by the publisher.

## References

[1] Juhaeri J Gao S Dai WS. Incidence Rates of Heart Failure, Stroke, and Acute Myocardial Infarction Among Type 2 Diabetic Patients Using Insulin Glargine and Other Insulin. Pharmacoepidemiol Drug Saf (2009) 18(6):497–503. doi: 10.1002/pds.1741 19326365

[2] Lara-Rojas CM Pérez-Belmonte LM López-Carmona MD Guijarro-Merino R Bernal-López MR Gómez-Huelgas R. National Trends in Diabetes Mellitus Hospitalisation in Spain 1997–2010: Analysis of Over 5. 4 Millions Admissions Eur J Intern Med (2019) 60:83–9. doi: 10.1016/j.ejim.2018.04.005 30100217

[3] Pérez-Belmonte LM Lara-Rojas CM López-Carmona MD Guijarro-Merino R Bernal-López MR Gómez-Huelgas R. National Trends in Heart Failure Hospitalization Rates in Patients With Diabetes Mellitus: 1997-2010. Rev Esp Cardiol (Engl Ed) (2018) 71(5):408–10. doi: 10.1016/j.rec.2017.04.028 28527562

[4] Seferovic PM Petrie MC Filippatos GS Anker SD Rosano G Bauersachs J. Type 2 Diabetes Mellitus and Heart Failure: A Position Statement From the Heart Failure Association of the European Society of Cardiology. Eur J Heart Fail (2018) 20(5):853–72. doi: 10.1002/ejhf.1170 29520964

[5] Davies MJ D’Alessio DA Fradkin J Kernan WN Mathieu C Mingrone G. Management of Hyperglycemia in Type 2 Diabetes, 2018. A Consensus Report by the American Diabetes Association (ADA) and the European Association for the Study of Diabetes (EASD). Diabetes Care (2018) 41(7):2669–701. doi: 10.2337/dc20-er07 PMC6245208 30291106

[6] Diabetes Canada Clinical Practice Guidelines Expert Committee Lipscombe L Booth G Butalia S Dasgupta K Eurich DT. Pharmacologic Glycemic Management of Type 2 Diabetes in Adults. Can J Diabetes (2018) 42:S88–103. doi: 10.1016/j.jcjd.2017.10.034 29650116

[7] Garber AJ Handelsman Y Grunberger G Einhorn D Abrahamson MJ Barzilay JI. Consensus Statement by the American Association of Clinical Endocrinologists and American College of Endocrinology on the Comprehensive Type 2 Diabetes Management Algorithm - 2020 Executive Summary. Endocr Pract (2020) 26(1):107–39. doi: 10.4158/CS-2019-0472 32022600

[8] Das SR Everett BM Birtcher KK Brown JM Januzzi JL Jr Kalyani RR. 2020 Expert Consensus Decision Pathway on Novel Therapies for Cardiovascular Risk Reduction in Patients With Type 2 Diabetes: A Report of the American College of Cardiology Solution Set Oversight Committee. J Am Coll Cardiol (2020) 76(9):1117–45. doi: 10.1016/j.jacc.2020.05.037 PMC7545583 32771263

[9] Pfeffer MA Claggett B Diaz R Dickstein K Gerstein HC Køber LV. Lixisenatide in Patients With Type 2 Diabetes and Acute Coronary Syndrome. N Engl J Med (2015) 373(23):2247–57. doi: 10.1056/NEJMoa1509225 26630143

[10] Marso SP Daniels GH Brown-Frandsen K Kristensen P Mann JF Nauck MA. Liraglutide and Cardiovascular Outcomes in Type 2 Diabetes. N Engl J Med (2016) 375(4):311–22. doi: 10.1056/NEJMoa1603827 PMC4985288 27295427

[11] Marso SP Bain SC Consoli A Eliaschewitz FG Jódar E Leiter LA. Semaglutide and Cardiovascular Outcomes in Patients With Type 2 Diabetes. N Engl J Med (2016) 375(19):1834–44. doi: 10.1056/NEJMoa1607141 27633186

[12] Holman RR Bethel MA Mentz RJ Thompson VP Lokhnygina Y Buse JB. Effects of Once-Weekly Exenatide on Cardiovascular Outcomes in Type 2 Diabetes. N Engl J Med (2017) 377(13):1228–39. doi: 10.1056/NEJMoa1612917 PMC9792409 28910237

[13] Hernandez AF Green JB Janmohamed S D’Agostino RB Sr Granger CB Jones NP. Albiglutide and Cardiovascular Outcomes in Patients With Type 2 Diabetes and Cardiovascular Disease (Harmony Outcomes): A Double-Blind, Randomised Placebo-Controlled Trial. Lancet (2018) 392(10157):1519–29. doi: 10.1016/S0140-6736(18)32261-X 30291013

[14] Husain M Birkenfeld AL Donsmark M Dungan K Eliaschewitz FG Franco DR. Oral Semaglutide and Cardiovascular Outcomes in Patients With Type 2 Diabetes. N Engl J Med (2019) 381(9):841–51. doi: 10.1056/NEJMoa1901118 31185157

[15] Gerstein HC Colhoun HM Dagenais GR Diaz R Lakshmanan M Pais P. Dulaglutide and Cardiovascular Outcomes in Type 2 Diabetes (REWIND): A Double-Blind, Randomised Placebo-Controlled Trial. Lancet (2019) 394(10193):121–30. doi: 10.1016/S0140-6736(19)31149-3 31189511

[16] Gerstein HC Sattar N Rosenstock J Ramasundarahettige C Pratley R Lopes RD. Cardiovascular and Renal Outcomes With Efpeglenatide in Type 2 Diabetes. N Engl J Med (2021) 385(10):896–907. doi: 10.1056/NEJMoa2108269 34215025

[17] Kristensen SL Rorth R Jhund PS Docherty KF Sattar N Preiss D. Cardiovascular, Mortality, and Kidney Outcomes With GLP-1 Receptor Agonists in Patients With Type 2 Diabetes: A Systematic Review and Meta-Analysis of Cardiovascular Outcome Trials. Lancet Diabetes Endocrinol (2019) 7(10):776–85. doi: 10.1016/S2213-8587(19)30249-9 31422062

[18] Valente MA Hillege HL Navis G Voors AA Dunselman PH van Veldhuisen DJ. The Chronic Kidney Disease Epidemiology Collaboration Equation Outperforms the Modification of Diet in Renal Disease Equation for Estimating Glomerular Filtration Rate in Chronic Systolic Heart Failure. Eur J Heart Fail (2014) 16(1):86–94. doi: 10.1093/eurjhf/hft128 23901055

[19] Comín-Colet J Garin O Lupón J Manito N Crespo-Leiro MG Gómez-Bueno M. Validation of the Spanish Version of the Kansas City Cardiomyopathy Questionnaire. Rev Esp Cardiol (2011) 64(1):51–8. doi: 10.1016/j.recesp.2010.10.003 21194819

[20] Zinman B Wanner C Lachin JM Fitchett D Bluhmki E Hantel S. Empagliflozin, Cardiovascular Outcomes, and Mortality in Type 2 Diabetes. N Engl J Med (2015) 373(22):2117–28. doi: 10.1056/NEJMoa1504720 26378978

[21] Neal B Perkovic V Mahaffey KW de Zeeuw D Fulcher G Erondu N. Canagliflozin and Cardiovascular and Renal Events in Type 2 Diabetes. N Engl J Med (2017) 377(7):644–57. doi: 10.1056/NEJMoa1611925 28605608

[22] Wiviott SD Raz I Bonaca MP Mosenzon O Kato ET Cahn A. Dapagliflozin and Cardiovascular Outcomes in Type 2 Diabetes. N Engl J Med (2019) 380(4):347–57. doi: 10.1056/NEJMoa1812389 30415602

[23] Cannon CP Pratley R Dagogo-Jack S Mancuso J Huyck S Masiukiewicz U. Cardiovascular Outcomes With Ertugliflozin in Type 2 Diabetes. N Engl J Med (2020) 383(15):1425–35. doi: 10.1056/NEJMoa2004967 32966714

[24] Bhatt DL Szarek M Steg PG Cannon CP Leiter LA McGuire DK. Sotagliflozin in Patients With Diabetes and Recent Worsening Heart Failure. N Engl J Med (2021) 384(2):117–28. doi: 10.1056/NEJMoa2030183 33200892

[25] McMurray JJ Solomon SD Inzucchi SE Køber L Kosiborod MN Martinez FA. Dapagliflozin in Patients With Heart Failure and Reduced Ejection Fraction. N Engl J Med (2019) 381(21):1995–2008. doi: 10.1056/NEJMoa1911303 31535829

[26] Packer M Anker SD Butler J Filippatos G Pocock SJ Carson P. Cardiovascular and Renal Outcomes With Empagliflozin in Heart Failure. N Engl J Med (2020) 383(15):1413–24. doi: 10.1056/NEJMoa2022190 32865377

[27] Alkhezi OS Alsuhaibani HA Alhadyab AA Alfaifi ME Alomrani B Aldossary A. Heart Failure Outcomes and Glucagon-Like Peptide-1 Receptor Agonists: A Systematic Review of Observational Studies. Prim Care Diabetes (2021) 15(5):761–71. doi: 10.1016/j.pcd.2021.04.005 33926837

[28] Velez M Peterson EL Wells K Swadia T Sabbah HN Williams LK. Association of Antidiabetic Medications Targeting the Glucagon-Like Peptide 1 Pathway and Heart Failure Events in Patients With Diabetes. J Card Fail (2015) 21(1):2–8. doi: 10.1016/j.cardfail.2014.10.012 25451709 PMC4276467

[29] Sardu C Paolisso P Sacra C Santamaria M de Lucia C Ruocco A. Cardiac Resynchronization Therapy With a Defibrillator (CRTd) in Failing Heart Patients With Type 2 Diabetes Mellitus and Treated by Glucagon-Like Peptide 1 Receptor Agonists (GLP-1 RA) Therapy vs. Conventional Hypoglycemic Drugs: Arrhythmic Burden, Hospitalizations for Heart Failure, and CRTd Responders Rate. Cardiovasc Diabetol (2018) 17(1):137. doi: 10.1186/s12933-018-0778-9 30348145 PMC6196445

[30] McDonagh TA Metra M Adamo M Gardner RS Baumbach A Böhm M. 2021 ESC Guidelines for the Diagnosis and Treatment of Acute and Chronic Heart Failure. Eur Heart J (2021) 42(36):3599–726. doi: 10.1093/eurheartj/ehab368 34447992

[31] Anker SD Butler J Filippatos GS Jamal W Salsali A Schnee J. Evaluation of the Effects of Sodium-Glucose Co-Transporter 2 Inhibition With Empagliflozin on Morbidity and Mortality in Patients With Chronic Heart Failure and a Preserved Ejection Fraction: Rationale for and Design of the EMPEROR-Preserved Trial. Eur J Heart Fail (2019) 21(10):1279–87. doi: 10.1002/ejhf.1596 31523904

[32] He Z Yang L Nie Y Wang Y Wang Y Niu X. Effects of SGLT-2 Inhibitors on Health-Related Quality of Life and Exercise Capacity in Heart Failure Patients With Reduced Ejection Fraction: A Systematic Review and Meta-Analysis. Int J Cardiol (2021) 345:83–8. doi: 10.1016/j.ijcard.2021.10.008 34653575

[33] Nielsen R Jorsal A Tougaard RS Rasmussen JJ Schou M Videbaek L. The Impact of the Glucagon-Like Peptide-1 Receptor Agonist Liraglutide on Natriuretic Peptides in Heart Failure Patients With Reduced Ejection Fraction With and Without Type 2 Diabetes. Diabetes Obes Metab (2020) 22(11):2141–50. doi: 10.1111/dom.14135 32627271

[34] Górriz JL Soler MJ Navarro-González JF García-Carro C Puchades MJ D’Marco L. GLP-1 Receptor Agonists and Diabetic Kidney Disease: A Call of Attention to Nephrologists. J Clin Med (2020) 9(4):947. doi: 10.3390/jcm9040947 PMC7231090 32235471

[35] Ussher JR Greenwell AA Nguyen MA Mulvihill EE. Cardiovascular Effects of Incretin-Based Therapies: Integrating Mechanisms With Cardiovascular Outcome Trials. Diabetes (2022) 71(2):173–83. doi: 10.2337/dbi20-0049 PMC8914293 35050311

[36] Greco EV Russo G Giandalia A Viazzi F Pontremoli R De Cosmo S. GLP-1 Receptor Agonists and Kidney Protection. Medicina (Kaunas) (2019) 55(6):233. doi: 10.3390/medicina55060233 PMC6630923 31159279

[37] Thomas MC. The Potential and Pitfalls of GLP-1 Receptor Agonists for Renal Protection in Type 2 Diabetes. Diabetes Metab (2017) 43:2S20–27. doi: 10.1016/S1262-3636(17)30069-1 28431667

[38] Nauck MA Meier JJ Cavender MA Abd El Aziz M Drucker DJ. Cardiovascular Actions and Clinical Outcomes With Glucagon-Like Peptide-1 Receptor Agonists and Dipeptidyl Peptidase-4 Inhibitors. Circulation (2017) 136(9):849–70. doi: 10.1161/CIRCULATIONAHA.117.028136 28847797

[39] Ramírez E Picatoste B González-Bris A Oteo M Cruz F Caro-Vadillo A. Sitagliptin Improved Glucose Assimilation in Detriment of Fatty-Acid Utilization in Experimental Type-II Diabetes: Role of GLP-1 Isoforms in Glut4 Receptor Trafficking. Cardiovasc Diabetol (2018) 17(1):12. doi: 10.1186/s12933-017-0643-2 29325553 PMC5765634

[40] Torekov SS. Glucagon-Like Peptide-1 Receptor Agonists and Cardiovascular Disease: From LEADER to EXSCEL. Cardiovasc Res (2018) 114(10):e70–1. doi: 10.1093/cvr/cvy124 30052921

[41] Akawi N Checa A Antonopoulos AS Akoumianakis I Daskalaki E Kotanidis CP. Fat-Secreted Ceramides Regulate Vascular Redox State and Influence Outcomes in Patients With Cardiovascular Disease. J Am Coll Cardiol (2021) 77(20):2494–513. doi: 10.1016/j.jacc.2021.03.314 PMC8141611 34016263

[42] Engelbrechtsen L Lundgren J Wewer Albrechtsen NJ Mahendran Y Iepsen EW Finocchietto P. Treatment With Liraglutide may Improve Markers of CVD Reflected by Reduced Levels of Apob. Obes Sci Pract (2017) 3(4):425–33. doi: 10.1002/osp4.133 PMC5729494 29259801

[43] Lundgren JR Færch K Witte DR Jonsson AE Pedersen O Hansen T. Greater Glucagon-Like Peptide-1 Responses to Oral Glucose are Associated With Lower Central and Peripheral Blood Pressures. Cardiovasc Diabetol (2019) 18(1):130. doi: 10.1186/s12933-019-0937-7 31586493 PMC6778378

[44] Lundgren JR Janus C Jensen SBK Juhl CR Olsen LM Christensen RM. Healthy Weight Loss Maintenance With Exercise, Liraglutide, or Both Combined. N Engl J Med (2021) 384(18):1719–30. doi: 10.1056/NEJMoa2028198 33951361

[45] Bell DSH Goncalves E. Heart Failure in the Patient With Diabetes: Epidemiology, Aetiology, Prognosis, Therapy and the Effect of Glucose-Lowering Medications. Diabetes Obes Metab (2019) 21(6):1277–90. doi: 10.1111/dom.13652 30724013

[46] Christensen RM Juhl CR Torekov SS. Benefit-Risk Assessment of Obesity Drugs: Focus on Glucagon-Like Peptide-1 Receptor Agonists. Drug Saf (2019) 42(8):957–71. doi: 10.1007/s40264-019-00812-7 30972641

[47] Makam AN Nguyen OK. An Evidence-Based Medicine Approach to Antihyperglycemic Therapy in Diabetes Mellitus to Overcome Overtreatment. Circulation (2017) 135(29):180–95. doi: 10.1161/CIRCULATIONAHA.116.022622 PMC5502688 28069712

[48] Pérez-Belmonte LM Ricci M Sanz-Cánovas J Cobos-Palacios L López-Carmona MD Ruiz-Moreno MI. De-Intensification of Antidiabetic Treatment Using Canagliflozin in Patients With Heart Failure and Type 2 Diabetes: Cana-Switch-HF Study. J Clin Med (2021) 10(9):2013. doi: 10.3390/jcm10092013 34066707 PMC8125841

